# Inositols in PCOS

**DOI:** 10.3390/molecules25235566

**Published:** 2020-11-27

**Authors:** Zdravko Kamenov, Antoaneta Gateva

**Affiliations:** Department of Internal Medicine, Clinic of Endocrinology University Hospital Alexandrovska, Medical University—Sofia, 1431 Sofia, Bulgaria; tony_gateva@yahoo.com

**Keywords:** PCOS, inositols, insulin resistance

## Abstract

(1) Background: Myoinositol (MI) and D-chiro-inositol (DCI) are involved in a number of biochemical pathways within oocytes having a role in oocyte maturation, fertilization, implantation, and post-implantation development. Both inositols have a role in insulin signaling and hormonal synthesis in the ovaries. (2) Methods: Literature search (with key words: inositols, myo-inositol, d-chiro-inositol, PCOS) was done in PubMed until Sept. 2020 and 197 articles were identified, of which 47 were of clinical trials (35 randomized controlled trials). (3) Results: Many studies have demonstrated that in patients with polycystic ovarian syndrome (PCOS) MI treatment improved ovarian function and fertility, decreased the severity of hyperandrogenism including acne and hirsutism, positively affected metabolic aspects, and modulated various hormonal parameters deeply involved in the reproductive axis function and ovulation. Thus treating with MI has become a novel method to ameliorate PCOS symptoms, improve spontaneous ovulation, or induce ovulation. The current review is focused on the effects of MI and DCI alone or in combination with other agents on the pathological features of PCOS with focus on insulin resistance and adverse metabolic outcomes. (4) Conclusions: The available clinical data suggest that MI, DCI, and their combination in physiological ratio 40:1 with or without other compound could be beneficial for improving metabolic, hormonal, and reproductive aspects of PCOS.

## 1. Overview of PCOS Pathogenesis

Polycystic ovarian syndrome (PCOS) is the most prevalent endocrine disorder in women of reproductive age, affecting approximately 6–15% of them [1,2,3]. It is a major cause of menstrual disturbances, hirsutism, and female anovulatory infertility [4]. However women with PCOS may also have other comorbidities including psychological (anxiety, depression, body image) [2,5,6], metabolic (obesity, insulin resistance, metabolic syndrome, prediabetes, type 2 diabetes, cardiovascular risk factors (hypertension, dyslipidemia), and increased risk for sleep apnea, endometrial carcinoma, and pregnancy-related complications (gestational diabetes, preeclampsia, pregnancy-induced hypertension, postpartum hemorrhage and infection, preterm delivery, meconium aspiration, stillbirth, operative deliveries, and shoulder dystocia) [7]. Thus PCOS negatively affects not only reproduction, but also general health, sexual health, and quality of life [3].

PCOS is currently diagnosed based on the Rotterdam criteria—presence of two out of three criteria—(1) oligo- or anovulation, (2) clinical and/or biochemical signs of hyperandrogenism, and (3) polycystic ovaries and exclusion of other etiologies (congenital adrenal hyperplasia, androgen-secreting tumors, Cushing’s syndrome) [8]. The use of Rotterdam criteria is advised by the two recent guidelines [7,9] both recognizing that Androgen Excess Society (AES) [1] criteria may correspond better to the pathogenesis of this disorder, as the AES emphasizes the importance of clinical and/or biochemical hyperandrogenism and placing less importance on polycystic ovarian morphology [9].

None of the diagnostic criteria include however the metabolic disturbances (insulin resistance and overweight/obesity), that are often found in PCOS patients and could play a crucial role in the pathogenesis of the syndrome [2,3,5,7,9].

The pathophysiological mechanisms by which PCOS negatively impacts fertility are complex and not completely understood. Undoubtedly, hyperandrogenism, the consequent hyperestrogenemia, insulin resistance, and compensatory hyperinsulinemia play an important role acting on both the ovary and the endometrium [10,11,12,13].

### 1.1. Polycystic Ovarian Syndrome and Insulin Resistance

Current evidence suggests that **insulin resistance** and compensatory hyperinsulinemia are a central feature of PCOS [14]. In most studies its prevalence is between 44% and 75% [15,16,17], which is much higher than 10–25% observed in young healthy individuals [18]. The patients with PCOS both with and without obesity have higher prevalence of insulin resistance compared to healthy controls [19], although insulin resistance is more severe in obese subjects [20]. According to our data there is also some differences in diagnostic value of different methods for diagnosing insulin resistance between lean and obese PCOS subjects [20].

There are many uncertainties about the exact reason for insulin resistance in PCOS. It is thought that insulin resistance in PCOS is endogenous but could be worsened by the presence of obesity. Morin-Papunen L.C. et al. confirm this hypothesis, although they fail to demonstrate decreased insulin sensitivity in lean PCOS patients [21]. Adipocytes, derived from obese PCOS patients do not show significantly decreased number and affinity of insulin receptors but decreased maximal glucose utilization and blunted inhibition of lipolysis by insulin are demonstrated [22]. Similar results are found in women with PCOS without obesity, impaired glucose tolerance, or increased waist-to-hip ratio (WHR), that support the endogenous genesis of insulin resistance [23].

Although the insulin resistance is thought to be fundamental for PCOS, **obesity** tends to worsen it and the accompanying metabolic disturbances [23,24] and contributes to the prevalence of the metabolic syndrome in PCOS patients [25]. The prevalence of obesity in PCOS varies between 30% and 70% depending on ethnicity and criteria for PCOS diagnosis [26,27]. Visceral obesity has a greater effect on insulin resistance than increased BMI per se and could be present even in PCOS patients with normal BMI [28,29].

**Figure 1:**
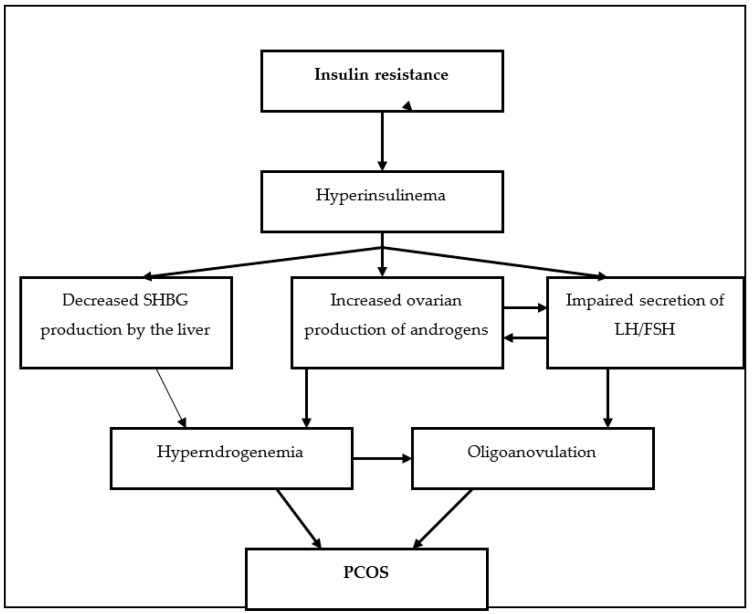
Role of insulin resistance in the development of polycystic ovarian syndrome (PCOS).

Biochemical hyperandrogenemia seen in PCOS shows positive correlation to the indices of insulin resistance in women with PCOS [30]. This implies a causal relationship between both conditions. The severity of hyperinsulinema directly correlates to the severity of PCOS [31,32], but it is uncertain whether hyperandrogenemia results from insulin resistance or vice versa or if both conditions are independently developed. Most authors however share the opinion that insulin resistance is the primary defect and hyperandrogenemia is secondary to that. Hyperinsulinemia appears to play an important pathogenic role in the hyperandrogenism and anovulation of both obese and lean women with PCOS [33,34]. Insulin can directly stimulate androgen secretion and/or increase lutein hormone (LH)-induced androgen secretion from the theca-cells [35,36], increase the amplitude of LH pulsatile secretion [34], decrease liver production of sex hormone binding globulin (SHBG) [37] (Figure 1). In ovarian granulosa and theca cells there are insulin receptors that mediate its metabolic, steroidogenic, and mitogenic actions [38].

Insulin resistance in PCOS however tends to be tissue selective. It affects muscle and fat tissue and the liver, but not the polycystic ovary itself [39,40]. Ovarian granulosa and theca cells, isolated form anovulatory PCOS patients show normal response to insulin [38,41,42]. In cell cultures they demonstrate normal secretion of estradiol and progesterone in response to physiologic concentrations of insulin, that implies normal insulin sensitivity [41,43]. Despite the insulin resistance in metabolic tissues, ovarian cells remain insulin sensitive, which leads to hyperinsulinemia-induced hyperandrogenemia in PCOS.

### 1.2. Polycystic Ovarian Syndrome and Sex-Hormonal Imbalance

**Hyperandrogenism** is a key diagnostic feature of PCOS affecting 60–100% of patients with the condition with both clinical (hirsutism, alopecia, and acne) and biochemical hyperandrogenism [7]. Hyperandrogenism induces chronic anovulation and menstrual disturbances in PCOS patients. Biochemically hyperandrogenemia is characterized by increased circulating levels of serum total and free testosterone and androstenedione and increased free androgen index (FAI). Currently there is enough data supporting the fact that increased androgen production is based on primary defect in steroid production from the theca-cells [10]. It is also possible that theca-cells in PCOS are more sensitive to gonadotropic hormones and produce increased amounts of androgens because of the effects of insulin and IGF-1. Assessments of free testosterone (FT) levels are more sensitive than the measurement of total testosterone (TT) for establishing the existence of androgen excess. The value of measuring the levels of androgens other than testosterone in patients with PCOS is relatively low. Although levels of dehydroepiandrosterone sulfate (DHEAS) are increased in about 30 to 35% of PCOS patients, its measurement does not add significantly to the diagnosis, and in the majority of the patients, free and total T are also increased [9].

The main neuroendocrine characteristic of PCOS is the **chronically increased frequency of the pulsatile secretion of GnRH** which leads to increased LH secretion and changes in LH/FSH ratio that are typical for this syndrome. Independently of their primary or secondary genesis neuroendocrine disturbances have a significant role in PCOS pathogenesis and progression. These disturbances are presented mainly as increased LH/FSH ratio. Many studies demonstrate elevated plasma levels of LH and decreased of FSH in 30% to 90% of the cases [44,45].

The factors regulating gonadotropic axis function in the hypothalamus are very complex and still not fully understood. In most mammals dopaminergic and opioidergic pathways inhibit GNRH-secreting neurons while noradrenergic pathways stimulate them. Tonic hypersecretion of LH in PCOS patients could be at least partially related to decreased dopaminergic and opioid tonus [46,47]. Increased insulin levels act synergistically with LH to stimulate ovarian production of androgens [38,48] and suppresses SHBG production from the liver [49], that leads to increased free testosterone levels. Although insulin resistance is well described as an important pathway of PCOS pathogenesis, its role for the neuroendocrine disturbances seen in those patients is still not fully understood. Insulin increases both basal and GNRH-stimulated secretion of LH and FSH in isolated pituitary cell cultures [50,51], but not in vivo [52]. Decreasing insulin levels on the other hand has no significant effect on LH secretion [53]. It is possible that PCOS-related hyperinsulinemia does not directly induce neuroendocrine disturbances, but its effect is rather indirect by increasing androgen levels.

### 1.3. Polycystic Ovarian Syndrome and Ovarian Changes

The presence of big **polycystic ovaries** with high antral (2–8 mm) follicle number is one of the main characteristics of PCOS [54]. This typical ovarian morphology results from the impaired follicular development and premature cessation of follicular growth subsequent to the endocrine disturbances including hyperandrogenemia, LH hypersecretion, and hyperinsulinemia. As a result, 60–80% of the patients have irregular menstruation, which is combined with anovulatory infertility [55]. There appear to be significant differences in metabolic profile between ovulatory and anovulatory patients with PCOS and hyperandrogenemia. Insulin resistance and hyperinsulinemia are more prevalent in anovulatory patients [32,45,56,57]. Lean patients with PCOS, oligo-anovulation, and hyperandrogenemia can have normal insulin sensitivity, but more frequently have increased LH levels than those with obesity [45,58]. It is possible that metabolic factors can worsen anovulation but they are usually added on to independent disturbances in folliculogenesis.

## 2. Inostols—Biological Role in Glucose Metabolism and Ovarian Function

**Inositols** are chemically identified as hexahydroxycyclohexanes and include a family of nine stereoisomers [59]. Myo-inositol (MI) is the most widely distributed in nature, including animals and mammals [60]. MI is ingested with food mostly from fruits, beans, grains, and nuts. Daily intake of MI from phytate-rich food does not exceed 500–700 mg/day for western diet. MI can also be actively synthetized (up to 4 g/day) in human body (especially the liver and brain) [61]. The cellular precursor of MI is glucose-6-phosphate, which is isomerized to inositol-3-phosphate (IP3) by D-3-myo-inositol-phosphate synthase. IP3 is then dephosphorylated to free MI by inositol monophosphatase-1. Free inositol may also be obtained by recycling inositol-1,4,5-trisphosphate and inositol1,4-bisphosphate [59]. MI biosynthesis varies among tissues depending on changing functional requirements.

There is a complex relationship between glucose and MI metabolism. On the one hand MI inhibits duodenal glucose absorption and reduces blood glucose rise, suggesting the existence of a competitive affinity for the same transporter system [59,62]. On the other hand glucose significantly counteracts cellular uptake of inositol and may induce MI depletion by the activation of the glucose-sorbitol pathway [63]. Inhibiting aldose reductase in cultured cells restores MI levels counteracting the depleting effect of sorbitol [63]. Inhibitors of the sodium-glucose transporters (SGLT) 1/2 prevent both glucose and inositol uptake [64], suggesting that the two molecules share the transporter system(s) [59]. In addition, both hyperglycemia and insulin resistance have been found to modify the relative ratio in which different inositol isomers are present in these tissues.

**Figure 2:**
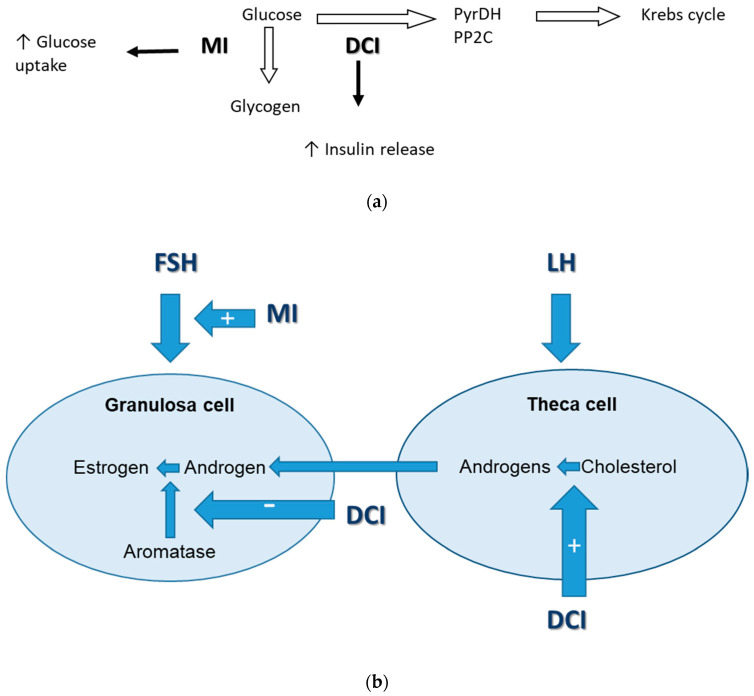
(**a**) Effects of myoinositol and D-chiroinositol on glucose metabolism in PCOS (adapted from [68]). (**b**) Effects of myoinositol and D-chiroinositol on hormonal synthesis in PCOS.

MI and DCI have a significant role in glucose metabolism—MI participates in the cellular uptake of glucose, inducing the GLUT4 translocation to cell membrane [65]; inhibits the adenylate cyclase enzyme; and reduces the release of free fatty acids from adipose tissues, while DCI participates in glycogen synthesis [66] (Figure 2). Both MI and DCI show insulin-mimetic properties and decrease postprandial blood glucose while glucose metabolism is shifted toward glycogen synthesis by DCI, and toward glucose catabolism by MI [67].

The inositol of the isomers, **myoinositol (MI) and D-chiroinositol (DCI),** are abundant in the ovaries and follicular fluid and have specific roles in insulin signaling and follicular development. MI stimulates FSH signaling as a second messenger, while DCI is responsible for insulin-mediated androgen synthesis and can act as an aromatase inhibitor. In the normal ovary the balance between those two isomers supports the normal hormonal secretion and ovarian function. Under physiological conditions, the MI/DCI ratio is between 100:1 in the follicular fluid and 40:1 in plasma [69,70]. In patients with PCOS and insulin resistance, hyperinsulinemia induces higher DCI-to-MI ratio because of stimulated epimerase activity, which transforms MI to DCI [69]. Despite the chemical similarities of MI and DCI and their synergistic effect on insulin sensitivity they exert different functions on the ovary. MI can affect aromatase activity in an opposite manner with respect to DCI [71]. In this way higher MI/DCI ratios promote the activity of aromatase in granulosa increasing estrogen levels, while lower MI/DCI ratios stimulate androgen production in theca cells [71]. This can explain why DCI supplementation produces an increase in testosterone levels and concomitant reduction of estrogens [72]. This leads to hyperandrogenism and suppressed FSH signaling. This mechanism is involved in the so called “ovarian paradox,” defined in 2011 by Carlomagno et al. who hypothesize that in the ovaries of polycystic ovary syndrome patients, enhanced epimerase activity leads to a local MI deficiency which in turn is responsible for the poor oocyte quality [73]. In these conditions, glucose uptake and metabolism in oocytes and follicular cells are negatively affected, thereby compromising oocyte quality that depends on the availability of adequate amounts of MI. This paradox is further supported by later animal and human studies that show differential effect of supplementation with MI only and MI + DCI in different ratios, proving that restoring physiological levels of the two inositol isomers could be crucial for proper ovarian function.

The various beneficial effects of inositols on follicular development, hormonal regulation, and glucose homeostasis support their use as therapeutic agents in patients with PCOS. Many studies confirm their positive effect on metabolic, hormonal, and reproductive disturbances in PCOS alone or in combination with other substances, enhancing their therapeutic effect and bioavailability. On the other hand MI treatment is safe and with very few side effects compared to other ovulation-induction therapeutic options. In 2015 International Consensus Conference on MI and DCI in Obstetrics and Gynecology recognizes that both MI and DCI are involved in several biological pathways, involved in PCOS pathogenesis and there is plenty clinical data demonstrating that inositols supplementation could be beneficial for improving metabolic and reproductive aspects of this disorder [74]. In recent years, several studies have proved their effectiveness in patients with PCOS [75].

## 3. Inositols and Insulin Resistance in PCOS

A crucial effect exerted by MI and DCI in PCOS patients is the insulin sensitizing action, which improves insulin resistance, mirrored by the homeostatic model assessment (HOMA-IR) index decrease [67].

Nestler et al. [68] were the first to report the efficacy of D-chiro-inositol in the treatment of obese PCOS women, demonstrating increased insulin action, improved ovulatory function, and decreased serum androgen concentrations, blood pressure, and plasma triglyceride concentrations. Few years later the same effects were demonstrated in lean PCOS women [76], where the area under the plasma insulin curve after oral administration of glucose decreased significantly and serum-free testosterone concentration decreased by 73% in comparison with essentially no change in the placebo group.

In later studies, treatment with MI proved its effectiveness in reducing hormonal, metabolic, and oxidative abnormalities in PCOS patients by improving insulin resistance [77]. Zacche et al. [78] showed a reduction of HOMA index from 2.9 ± 0.8 to 1.4 ± 0.5 (*p* < 0.01) in PCOS patients after three months of MI treatments and A. Genazzani et al. [79] demonstrated the same effect in overweight PCOS women —HOMA reduction from 2.8 ± 0.6 to 1.4 ± 0.3 (*p* < 0.01), while Minozzi et al. [80] showed a reduction from 2.9 ± 0.9 to 1.8 ± 1.0 (*p* < 0.05) after 12 months treatment with a combination of MI and combined oral contraceptive (COC), that was significantly more effective than oral contraceptive alone. The beneficial effect of MI on insulin sensitivity was confirmed in other studies [81], where plasma insulin levels, glucose-to-insulin ratio, and HOMA index significantly improved after 12 weeks of treatment in combination with reduction of plasma LH, prolactin, testosterone levels, and LH/FSH ratio. Improved insulin resistance and ovulatory function was also seen after combined MI + DCI treatment [82].

A recent meta-analysis [83] that evaluated the efficacy of treatments with MI, alone or combined with DCI (40:1 ratio between MI and DCI) for 12–24 weeks, in nine randomized controlled trials (RCTs) comprising 247 cases and 249 controls showed significant reductions in fasting insulin (standardized mean difference = −1.021 μU/mL, 95% CI: −1.791 to −0.251, *p* = 0.009) and HOMA-IR index (standardized mean difference = −0.585, 95% CI: −1.145 to −0.025, *p* = 0.041) after inositol supplementation.

Recently it has been shown that similarly to metformin MI restores the diminished GLUT-4 protein levels and glucose uptake through sodium-myoinositol cotransporter-1 (SMIT-1) and p- AMP-activated (AMPK)-dependent mechanism [67]. A meta-analysis of 10 randomized controlled studies (6 RCTs compared MI with placebo, one RCT compared MI + D-chiro-inositol with placebo and 3 studies compared MI with metformin or COC) further confirmed the beneficial effects of inositols on insulin sensitivity showing significantly improved HOMA index (weighted mean difference = −0.65; 95% CI: −1.02, −0.28; *p* = 0.0005) and raised E2 levels (weighted mean difference = 16.16; 95% CI: 2.01, 30.31; *p* = 0.03) and only a trend in reducing total testosterone levels [84].

A more recent study that compares inositols to other treatment strategies (COC and metformin) shows that inositol therapies (either MI + folic acid or MI + DCI + folic acid) improved significantly insulin resistance and glycosylated hemoglobin, reducing cholesterol and triglyceride levels and blood pressure (when used for more than 3 months), while COC treatment worsened insulin resistance and lipid parameters (increasing cholesterol and triglyceride levels), reducing significantly at the same time FSH, LH, and SHBG serum levels [85].

Metformin is an antidiabetic drug that is not only a first choice treatment of type 2 diabetes mellitus but because of its proven insulin-sensitizing properties has been also widely used in other conditions associated with insulin resistance, including PCOS. It acts on different tissues and reduces glycemia levels, decreases the ovarian production of androgens and the concentration of circulating androgens, and improves ovulatory function. A recent meta-analysis of six clinical trials, with a total of 355 patients, treated ether with metformin (*n* = 178) or inositols (*n* = 177) demonstrates similar effect of metformin and MI on fasting insulin, HOMA index, testosterone, androstenedione, SHBG, and body mass index (BMI) with less side effects registered in patients taking MI compared to metformin (RR = 5.17; 95% CI: 2.91–9.17; *p* < 0.001) [86].

In another study the effect of MI on fasting plasma glucose serum insulin levels, serum triglyceride, and VLDL-cholesterol levels and quantitative insulin sensitivity check index was significantly higher compared with metformin. MI supplementation also upregulated gene expression of peroxisome proliferator-activated receptor gamma (PPAR-γ) (*p* = 0.002) compared with metformin [87]. The authors highlighted that MI exerts its therapeutic activity by means of PPAR-gamma activation, without affecting GLUT-1 and LDLR gene expression.

MI and metformin in combination could act in an additive or synergistic way allowing the use of reduced doses of metformin in patients intolerant to the normal therapeutic administration of metformin. Confirming this hypothesis the combination metformin + MI showed greater reduction of HOMA-IR at 3 months of treatment (*p* = 0.03) than metformin alone while the effect of fasting blood glucose and insulin levels was not statistically different [88]. Combination treatment also lead to greater improvement in menstrual cycles (both length and bleeding per cycle), BMI, acne score, and modified Ferriman Gallway score and hormonal parameters improved in both the groups and the levels were comparable after 3 months.

## 4. Inositols and Other Metabolic Abnormalities in PCOS

While some studies find significantly decreased BMI following MI treatment [89,90,91,92,93,94,95], A. Genazzani et al. [91] showed a nonsignificant change of BMI, although improvement of other PCOS-related parameters was improved. After 12 months of combined treatment with MI and combined oral contraceptive (COC) there also was no significant difference in BMI [82], despite the greater effect on endocrine, metabolic, and clinical profile in patients with PCOS than oral contraceptive alone. In another study the treatment with oral contraceptive led to slightly increased weight and BMI with no effect on metabolic parameters, while the combination MI + COC did not induce any changes in weight and BMI [87].

In morbidly obese patients (BMI > 37 kg/m^2^) some of the beneficial effects of MI were blunted and there was an inverse relationship between BMI and treatment [90]. Other studies however show opposite results and demonstrate that MI administration is more effective in obese patients with high fasting insulin plasma levels [91].

Some studies also demonstrate that the combination of MI and D-chiro-inositol in their physiological plasma ratio 40:1 is more effective than MI alone also in reducing LDL-cholesterol, triglycerides and HOMA-index [92,93]. A recent study on 43 overweight and obese PCOS patients divided into three groups and treated for six months: group 1 (*n* = 21) with diet (1200 Kcal) only; group 2 (*n* = 10) with diet plus MI (2 g MI and 200 μg folic acid in powder, twice daily); group 3 (*n* = 12) with diet associated to MI and DCI in the 40:1 ratio (2 soft gel capsules, containing 550 mg MI, 13.8 mg DCI and 200 μg folic acid, per day) showed a significant decrease in weight, BMI, waist and hip circumferences decreased significantly in all the patients [94]. The addition of MI plus DCI to the diet seems to accelerate the improvement of weight and fat mass, with a slight increase of lean mass. The three groups did not show any significant difference regarding the improvement of the Ferriman–Gallway score. Instead, the patients significantly differed with regard to the restoration of menstrual regularity.

Another study with different MI:DCI ratio, 10:1 administered for 6 months also showed significant body weight reduction and decreases in blood glucose-free testosterone, FSH, LH, and insulin levels, as well as significant increase of serum SHBG concentrations [95].

## 5. Inositols and Hyperandrogenism in PCOS

In patients with mild and moderate hirsutism the administration of 2g MI twice daily for 6 months led to significant decrease in the severity of hirsutism and the levels of total androgens, FSH, LH, and LDL cholesterol [96]. In another study plasma LH, prolactin, testosterone, insulin levels, and LH/FSH were significantly reduced and insulin sensitivity was significantly improved after 12 weeks of treatment with MI. The Ferriman–Gallway score decreased although the reduction was not statistically significant (22.7 ± 1.4 to 18.0 ± 0.8) whereas the reduction of the ovarian volumes was significant (12.2 ± 0.6 mL to 8.7 ± 0.8 mL, *p* < 0.05) [79]. This was confirmed by Ozay A. et al. who demonstrate that fasting glucose, LDL, DHEAS, total cholesterol, and prolactin levels decreased significantly in MI + folic acid-treated patients [97].

Beneficial effects on hormonal profile (decreased free testosterone and LH levels and increased estradiol and SHBG) were also observed after administration of MI + DCI in a 40:1 ratio, compared to those given a placebo [82].

Besides the beneficial effects on insulin sensitivity the meta-analysis by Unfer et al. [83] also demonstrated slight trend toward testosterone decrease with respect to controls, whereas androstenedione levels remained unchanged. MI was also able to significantly increase SHBG levels after at least 24 weeks of administration (standardized mean difference = 0.425 nmol/L, 95% CI: 0.050–0.801, *p* = 0.026).

A more recent study, conducted by Regidor et al. demonstrated changes in the testosterone level from 96.6 ng/mL to 43.3 ng/mL, and in the progesterone level from 2.1 ng/mL to 12.3 ng/mL, after MI and folic acid treatment in patients with PCOS [98].

## 6. Inositols and Menstrual Disturbances/Ovulation in PCOS

Some studies have demonstrated that MI treatment in patients with PCOS improved ovarian function and fertility [98,99,100,101,102,103,104,105,106,107,108,109], decreased the severity of hyperandrogenism, acne, and hirsutism [78,96,102], and positively affected metabolic parameters and modulated various hormonal parameters deeply involved in the reproductive axis function and ovulation [71,103] and thus it became a novel method to improve spontaneous ovulation [79,90,99] or ovulation induction [104,105,106].

In a study by Papaleo [99] there was a beneficial effect on restoration and maintenance of normal menstrual cycle during 6 months of MI treatment. Similar results were shown in another study that demonstrated significantly higher ovulation frequency in the MI-treated group (25%) with shorter time to first ovulation compared with the placebo (15%) [94]. Raffone reported that 65% of MI-treated patients restored spontaneous ovulation activity, compared to 50% of metformin-treated patients [101]. Gerli et al. [107] showed that ovulation frequency was significantly higher (*p* < 0.01) in MI-treated group (23%) compared with placebo (13%). E2 concentration increased only in MI group during the first week of treatment inducing follicular maturation. In another study ovulation was restored in 69.5% of women in MI group and 21% of placebo (*p* = 0.001). After treatment, the peak level of progesterone was higher in MI patients (15.1 ± 2.2 ng/mL) compared to placebo [108]. In another study progesterone and AMH levels, ovarian volume, ovarian antral follicle, and total antral follicle counts decreased significantly both in MI and combined contraceptive-treated patients with PCOS [97]. In an observational study performed in 3602 infertile women with PCOS MI + folic acid treatment for mean 10.2 weeks resulted in restoration of ovulation in 70% of women and 545 pregnancies (15.1% of all MI treated patients) [109].

The combination of MI and metformin showed better effect on menstrual cycle than metformin alone despite the similar effect of both treatments on weight, BMI, waist and hip circumferences [110].

Compared to clomiphene citrate MI showed nonsignificant trend to lower the resistance rate, lower ovulation rate, and higher pregnancy rate. The rate of multiple pregnancy was 18.1% in clomiphene group and 0% in the MI group [105]. In a study by Kamenov Z. et al. [104] MI monotherapy resulted in 61.7% ovulation rate (of those 37.9% became pregnant) during three spontaneous menstrual cycles. In the MI-resistant patients combination of MI and clomiphene citrate was used in the next three cycles and on this combination 72.2% ovulated (42.6% of those became pregnant). MI supplementation also produced very good clinical results with a significant reduction in cancellation rate (0 vs. 40%) and improvement in clinical pregnancy rate in patients with PCOS and insulin resistance, undergoing gonadotropin ovulation induction [106].

In the experimental (mice) model of PCOS, MI/DCI treatment in a 40:1 ratio made a fast recovery from PCOS signs and symptoms, while other MI/DCI ratios were less effective or had even negative effects [111]. This was confirmed recently by a human study in PCOS women where the ratio 40:1 was most effective in restoring ovulation and normalizing important parameters in these patients while decreased activity was observed with other formulations (1:3.5; 2.5:1; 5:1; 20:1; 80:1), especially when the 40:1 ratio was modified in favor of DCI [112]. Some studies however show different results—the combination 550 mg MI + 150 mg DCI twice daily (3:1) showed higher pregnancy and live birth rates and lower risk of ovarian hyperstimulation syndrome (OHSS) compared to the control group (CG) was administered 550 mg of MI + 13.8 mg of DCI twice daily (40:1) [113].

A more recent study in a small patient cohort however showed that the combination 550 mg of MI + 300 mg of DCI daily (≈2:1) had a positive influence on pregnancy rate (4 vs 1, *p* = 0.036), cytoplasm, perivitelline space, plasma membrane, and cone injection compared to lower dose combination—550 mg of MI with 27.6 mg of DCI (20:1). According to a multivariate analysis using linear mixed effect models, high doses of DCI have a positive influence on the “cytoplasm” (b = 1.631, v2 value = 7.42, d.f. = 1, *p* = 0.00645) [114].

The decreased efficacy found in PCOS treatment in some studies when patients were administered high doses of DCI may be explained by different biological mechanisms. It has been found that intestinal absorption of MI is reduced by the simultaneous administration of DCI since the two stereoisomers compete with each other for the same transporter that has similar affinity for each of them. On the other hand DCI was found to be an aromatase inhibitor which increases androgens and may have harmful consequences for normal ovulatory function [115].

The main effects of inositols on metabolic abnormalities, hyperandrogenism and menstrual cyclicity/ovulation are shown on Table 1.

## 7. Inositols and Gestational Diabetes in PCOS

Polycystic ovary syndrome is a primary risk factor for adverse pregnancy outcomes [66]. A meta-analysis conducted by Kjerulff et al. [116] indicated that pregnancy in PCOS patients is associated with increased risk of gestational diabetes (GDM), pregnancy-induced hypertension, preeclampsia, and other pregnancy-related complications and thus PCOS is an established risk factor for gestational diabetes [117].

Some studies show that MI decreased the risk of gestational diabetes by 50–67% among women with a family history of type 2 diabetes, as well as among women who were overweight and obese [118,119,120].

There are few studies that show a possible role of MI for primary prevention of gestational diabetes mellitus (GDM) in PCOS patients. In a study by D’Anna R. et al. MI was given through the whole pregnancy and a group of women treated with metformin stopped the drug after pregnancy diagnosis, and was considered as the control group. Prevalence of GDM in the MI group was 17.4% versus 54% in the control group, with a highly significant difference. Consequently, in the control group the risk of GDM occurrence was more than double compared to the MI group, with an odds ratio 2.4 (confidence interval 95%, 1.3–4.4) [121]. In patients with already diagnosed gestational diabetes there was an improvement in glucose homeostasis measurements (fasting glucose and insulin, and consequently HOMA index) after MI treatment [122]. The rate of preterm birth and gestational hypertension remained unchanged.

## 8. Inositols in Combination with Other Compounds in PCOS Treatment

In the treatment of PCOS, inositols are most commonly used in combination with **folic acid**. The trials assessing this combination show improved reproductive [100], hormonal, and metabolic disturbances in PCOS patients [95,97,108,123].

Recent therapeutic evidence addressed the importance of the association between **alpha lipoic acid** and MI or DCI. a-Lipoic acid (ALA) is a powerful antioxidant and enzymatic cofactor of the mitochondrial respiratory chain, in turn, capable of increasing insulin sensitivity. It is believed to directly scavenge ROS and reactive nitrogen species (RNS), both in vitro and in vivo. Both MI and ALA have an independent role in the activation of GLUT-4 crucial for glucose uptake from the cells [124] and thus can probably exert pleiotropic effects on carbohydrate metabolism.

The combination between DCI and ALA did not significantly improve the clinical and metabolic disturbances in PCOS patents [125], although other studies show some benefits of ALA on glucose uptake in lean PCOS patients [126]. One study that evaluated the effects of the combination of DCI and alpha lipoic acid (1000 mg DCI and 600 mg alpha lipoic acid daily) on menstrual cycle and insulin sensitivity in 41 women with polycystic ovary syndrome and 31 controls found that the association of DCI and alpha lipoic acid improves menstrual cycle length, restoring ovulation in the majority of women [127]. During treatment, BMI significantly decreased from 26.2 ± 5.3 (95% CI: 24.7–27.3) to 25.1 ± 5.2 (95% CI: 23.5–26.4) (*p* < 0.002) while HOMA-index decreased from 2.6 ± 2.0 (95% CI: 2.21–3.07) to 2.2 ± 1.6 (95% CI: 1.96–2.53) but without reaching statistical significance (*p* < 0.22). However, when the patients were divided based on the presence of insulin resistance (IR) (HOMA >2.5), HOMA-index decreased significantly from 4.7 ± 2.1 (95% CI: 3.24–5.70) to 3.2 ± 2.1 (95% CI: 2.02–4.23) (*p* < 0.05) only in IR group, remaining unchanged in the No-IR group. A recent study demonstrated that in overweight/obese PCOS patients with diabetic relatives undergoing IVF the combination of DCI 500 mg and ALA 300 mg leads to lower dose of gonadotropin, shorter stimulation days, higher number of MII oocytes, and higher number of fertilized oocytes [128].

In a small patient cohort (*n* = 40) De Cicco et al. [129] demonstrated that the combination of MI +ALA for 6 months decreased BMI, waist-hip ratio, hirsutism score, AMH, ovarian volume, and antral follicle count, and an increase in the number of menstrual cycles. A longer duration study with MI and ALA (2000 mg + 800 mg per day) demonstrated improvement in cycle length at 6 (*p* < 0.001), 12, and 24 months of treatment (*p* < 0.01), from a basal length of 69.25 ± 35.24 days, it was progressively reduced up to 34.89 ± 11.53 days after 24 months [130]. BMI showed a reduction in 75% of the patients only at 6 months (from 27.05 ± 4.17 kg/m^2^ to 25.36 ± 4.06 kg/m^2^, *p* < 0.05), thereafter returning similar to the basal values. After one year of treatment, no changes in hirsutism were reported by 53% of patients, an improvement by 31% and a worsening by 16% of women investigated. No changes of HOMA-IR, fasting insulin testosterone, and ovarian volume were observed, while insulin response to a 3 h OGTT was improved after 6 (*p* < 0.01) and 18 months (*p* < 0.05) of treatment.

Another study aimed to evaluate the effects of a treatment with α-lipoic acid (800 mg per day) in combination with two different doses of MI on clinical and metabolic features in 71 women with PCOS—43 patients received 2000 mg of MI and 28 received 1000 mg of MI per day [131]. Women with IR showed a significant reduction of BMI, fasting insulin, and of HOMA-IR (*p* < 0.01) and an increase of E2 (*p* < 0.05). Cycle length was improved in 80.0% of patients with IR and in 70.8% of those without IR (NS). The observed beneficial effects were more evident with the higher dose of MI. 85.7% of women taking 2000 mg of MI reported a higher improvement of menstrual regularity than those taking 1000 mg of MI (*p* < 0.01).

A recent retrospective study evaluated the effects of MI (1 g/day per os), alpha-lipoic acid (400 mg/day per os), and a combination of both in 90 overweight/obese patients with PCOS [132]. MI improved hormonal disturbances and insulin resistance mainly in PCOS patients with no familial diabetes, ALA improved insulin resistance and metabolic parameters in all patients with no effects on reproductive hormones, while MI + ALA combination improved hormonal and metabolic aspects and insulin response to OGTT in all patients. MI was less effective when familial diabetes was present, while MI + ALA combination was effective on all PCOS patients independent of familial diabetes.

In adolescent PCOS patients an elevated high-mobility-group-box-1 (HMGB1), associated with insulin resistance and inflammation, was demonstrated. After 6 month treatment with MI + ALA a significant reduction in HMGB1 level, serum insulin, HOMA-IR, and 17-hydroxyprogesterone was observed [133].

A recent study showed that the combination of insulin-sensitizing agents (MI, DCI, and chromium picolinate), **antioxidants,** and **vitamins** leads to improvements in menstrual cyclicity, acne, and hirsutism in both obese and lean PCOS patients [134]. Significant reduction in body weight was seen only in obese subjects.

Another combination that showed positive results on menstrual cycle, ovulation rate, and body weight in PCOS patients is 2 g myo-inositol, 0.5 mg **L-Tyrosine**, 0.2 mg **folic acid**, 55 mcg **selenium**, 40 mcg **chromium [135]**.

A pilot study showed a significant reduction in BMI, modest improvement in menstrual cycle regularity, and some changes in metabolomics profile following 3 months of combined treatment with MI (1.75 g/day), DCI (0.25 g/day), and **glucomannan** (a water-soluble fiber that is derived from the konjac root) (4 g/day) [136].

A novel combination, used mainly for treatment of reproductive disturbances in PCOS patients is the one with **melatonin**. Addition of melatonin seems to ameliorate the activity of MI and folic acid by improving oocyte quality and pregnancy outcome in women with low oocyte quality history [137]. This effect was confirmed in a more recent study, where MI and melatonin have shown to enhance synergistically oocyte and embryo quality [137]. Similar results were found when MI was combined with melatonin in the first 3 months before oocyte pick up and with vitamin D3 in the further 3 months, when significant improvements in blastocyst and oocyte quality were observed, achieving the 42% of clinical pregnancies vs. 24% in the control group, that received only 200 µg folic acid twice a day [138].

## 9. Resistance to Myoinositol in PCOS Patients

Despite the very good effect of MI on metabolic, hormonal, and reproductive parameters of PCOS patients, 25% to 75% of them could be resistant to this treatment. The reason for this resistance is still unclear but could be related to the state of obesity, insulin resistance, and hyperandrogenemia or differences in compound bioavailability. The cause of inositol resistance is not yet well understood. In most of the trials evaluating the rate of MI-induced ovulation the differences in terms of hormonal and metabolic profile between responders and non-responders were not assessed [139].

One study that compared 12 patients who responded to MI by establishing normal ovulation frequency (*n* = 6) and/or pregnancy (*n* = 6) with those patients who did not respond (less than three ovulations in 16 wk; *n* = 9) showed that the two groups presented with similar BMI, WHR, and circulating E2 and inhibin-B concentrations [90]. However, responders to MI treatment showed significantly lower testosterone levels (2.3 nmol/L vs. 3.4 nmol/L, respectively), higher SHBG (35.9 nmol/L vs. 25.8 nmol/L; *p* < 0.05), and thus lower free androgen index (6.9 vs. 11.6; *p* = 0.01). Fasting insulin or glucose concentrations or responses to the OGTT were not different. The authors conclude that the least androgenic patients were more likely to respond with establishment of normal menstrual rhythm to MI treatment.

In the study by Kamenov Z. et al. [104] most of the patients resistant to MI were obese. Compared to a normal weight woman, the obese had nearly the half probability to ovulate and a quarter the chance to become pregnant.

In a recent study by Olivia M et al. [140] MI-resistant patients did not show increased plasma levels of MI, raising the question about the role of MI bioavailability in non-responders. MI bioavailability is affected by many different factors, including intestinal absorption, transport from plasma into tissues, endogenous synthesis and catabolism, kidney excretion etc. In MI-resistant patients, who fail to ovulate on monotherapy, a few different agents could be added to improve ovulation and pregnancy rate (clomiphene, rFSH, lactalbumin).

**Clomiphene citrate** is an estrogen receptor modulator that blocks the negative feedback mechanism resulting in increased secretion of follicle-stimulating hormone (FSH). In a study by Kamenov Z. et al. [104] the MI-resistant patients (38.3%) were treated with a combination of MI and clomiphene citrate during the next three cycles and on this combination 13 of the 18 patients (72.2%) ovulated and five (10.6%) did not achieve ovulation. Six out of the 13 ovulatory women (42.6% or 12.8% of all women) became pregnant and seven (53.8% or 14.9% of all women) did not. The authors concluded that in non-ovulating or nonpregnant patients after MI treatment a combination with clomiphene citrate could be useful to achieve the goal of ovulation/pregnancy.

In a study by Raffone E. et al. [101] MI-treated patients who did not achieve pregnancy (*n* = 38) continued MI use and underwent ovulation induction with recombinant FSH for a maximum of three attempts. A very low-dose protocol (37.5 U/day) beginning from the day two of menstrual cycle in a step-up regime was selected. Pregnancy occurred in a total of 11 women (28.9%). Eight of these pregnancies occurred in the MI-resistant patients (*n* = 17), whereas three in the group which had ovulation restored with MI alone.

**α-lactalbumin** is a protein found in milk (20–25% of whey) that has a role not only as a nutrient but as a factor for resorption of other nutrients such as vitamins and microelements. In a very recent study (Olivia et al. 2018) 14 MI-resistant PCOS patients were treated with a combination of 2 MI plus 50 mg α-lactalbumin, twice a day, for three months. Among these 14 subjects, 12 (86%) ovulated. Their MI plasma levels at the end of the treatment significantly improved compared to the baseline (35.0 ± 3.8 μmol/L versus 17.0 ± 3.5 μmol/L,) and were similar to the patients who responded positively to the treatment with MI alone (38 ± 2.9 μmol/L). The addition of α-lactalbumin could play a beneficial role for MI bioavailability by changes in tight junctions permeability thus increasing plasma concentration in simultaneous administration [140].

## 10. Conclusions

The available clinical data suggest that myoinositol, D-chiro-inositol, and their combination in physiological ratio 40:1 with or without other compound could be beneficial for improving metabolic, hormonal, and reproductive aspects of PCOS.

## Figures and Tables

**Table 1:** Summary of the beneficial effects of myoinositol (MI), D-chiro-inositol (DCI), or the combination on both on metabolic, hormonal, and ovulatory disturbances in PCOS.

	Dosage	Effects on Metabolic Abnormalities	Effects on Hyperandrogenism	Effect on Menstrual Cyclicity/Ovulation
**MI**	1200–4000 mg daily [77,78,81]	Improved insulin sensitivity Reduced BMI	Decreased testosterone levels Decreased plasma LH levels and LH/FSH ratio Decreased FG score	Improved menstrual cycle Improved ovulation rate
**DCI**	1200 mg daily [68]	Improved insulin sensitivity Decreased BP Decreased TG concentrations	Decreased serum androgen concentrations	
**MI + DCI 40:1**	550 mg + 13.8 mg daily [92,93,94]	Improved insulin sensitivity Decreased BP Decreased TG and TChol concentrations Decreased BMI and fat mass	Decreased serum androgen concentrations Increased SHBG	Improved menstrual cycle Improved ovulation rate
**MI + DCI in other ratios**	10:1—500 mg + 50 mg [95] 0:1; 1:3.5; 2.5:1; 5:1; 20:1; 80:1 (total daily dose 2000 mg) [112] 2:1 (500 mg + 300 mg) [114] 3:1 (550 mg+150 mg)	Decreased blood glucose Decreased insulin levels Decreased BMI	Decreased testosterone levels Decreased FSH, LH levels Increased SHBG concentrations	Higher pregnancy rate positive effect on the “cytoplasm” Higher pregnancy and live birth rates Lower risk of ovarian hyperstimulation syndrome (OHSS)

BP—blood pressure, FG score—Ferriman–Galway score, TChol—total cholesterol, TG—triglycerides.
